# Basilar Artery Tortuosity Increases the Risk of Persistent Dizziness and Unsteadiness After Posterior Circulation Infarction

**DOI:** 10.1002/brb3.70097

**Published:** 2024-10-22

**Authors:** Jiashu Li, Xuesong Bai, Gaifen Liu, Zhaoxia Li, Yan Wang, Ruile Fang, Fei Peng, Xuge Chen, Yi Ju, Xingquan Zhao

**Affiliations:** ^1^ Department of Neurology, Beijing Tiantan Hospital Capital Medical University Beijing China; ^2^ China National Clinical Research Center for Neurological Diseases Beijing China; ^3^ Clinical Center for Vertigo and Balance Disturbance Capital Medical University Beijing China; ^4^ Beijing Neurosurgical Institute and Beijing Tiantan Hospital Capital Medical University Beijing China

**Keywords:** basilar artery tortuosity, dizziness, posterior circulation infarction, unsteadiness

## Abstract

**Background and Purpose:**

Basilar artery (BA) tortuosity is closely associated with posterior circulation infarction (PCI) and dizziness/unsteadiness. This study aims to determine the relationship between BA tortuosity and the outcome of dizziness and unsteadiness in PCI patients.

**Method:**

This study prospectively recruited PCI patients presenting with dizziness and unsteadiness. BA tortuosity was diagnosed based on Smoker's criteria. The BA tortuosity index (BATI) was measured from magnetic resonance angiography (MRA) images. Posterior circulation was divided into proximal (medulla oblongata and posterior inferior cerebellar), middle, and distal territories. Symptoms, risk of falls, and quality of life were followed up in 3 months after stroke. Logistic regression was used to identify possible factors associated with the persistence of dizziness and unsteadiness.

**Results:**

Among 182 PCI patients presenting with dizziness and unsteadiness, 97 (53.3%) had BA tortuosity, including 19 (10.4%) with moderate‐to‐severe BA tortuosity. At the 3‐month follow‐up, 58 (31.9%) patients continued to experience dizziness and unsteadiness, with significantly decreased quality of life and a high risk of falls. Binary logistic regression analysis identified moderate‐to‐severe BA tortuosity (OR, 4.474; 95% CI, 1.591*–*12.579; *p* = 0.004) and lesions involving the proximal posterior circulation territory (OR, 2.146; 95% CI, 1.097*–*4.199; *p* = 0.026) as risk factors for persistent dizziness and unsteadiness after PCI, while thrombolysis (OR, 0.280; 95% CI, 0.079*–*0.992; *p* = 0.049) as a protective factor. BATI (OR, 1.072; 95% CI, 1.028*–*1.119; *p* = 0.001) was also independently associated with dizziness and unsteadiness after PCI.

**Conclusion:**

Prominent BA tortuosity increases the risk of persistent dizziness and unsteadiness after PCI, leading to a high risk of falls and decreased quality of life. This warrants more attention in clinical practice.

## Graphical abstract

In this prospective study of 182 posterior circulation infarction (PCI) patients presenting with dizziness and unsteadiness, 31.9% reported persistent dizziness and unsteadiness at 3‐month follow‐up, associated with increased risk of fall and decreased quality of life. Prominent basilar artery tortuosity, proximal posterior circulation territory infarcts, and the absence of thrombolysis emerged as predictive factors. Targeted clinical management is essential for these high‐risk patients after PCI.

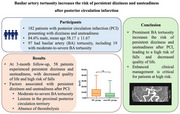

## 1 Introduction

The vertebrobasilar artery (VBA) serves as the primary blood supply to the posterior circulation. As a common morphological change observed in the basilar artery (BA), BA tortuosity is one of the main diagnostic criteria for vertebrobasilar dolichoectasia (VBD), a vasculopathy featuring the ectasia, elongation, and tortuosity of the VBA (Smoker et al. 1986). The clinical manifestations of VBD range from completely asymptomatic to benign or even malignant co‐morbidities, among which posterior circulation infarction (PCI) is the most common presentation (Passero and Rossi 2008). However, previous studies and clinical practice have found that a number of patients with simple BA tortuosity fail to meet the diagnostic criteria for VBD, but this clinical entity receives insufficient attention in clinical practice (Cao et al. 2023).

Vestibular symptoms such as dizziness and unsteadiness are the most common symptoms in PCI (Kim et al. 2022). Large prospective studies have reported that 47%–75% of PCI patients present with dizziness and nearly one third of them present with unsteadiness (Akhtar et al. 2009; Searls et al. 2012). These symptoms may relieve within a few weeks after stroke onset, while some patients may continue to experience dizziness and unsteadiness (Conrad et al. 2020). Persistent dizziness and unsteadiness can lead to an increased risk of falls, with more accompanied anxiety, depression, physical disability, and psychological stress, which strongly affects the life quality of patients (Curthoys and Halmagyi 1995; Balaban and Jacob 2001; Jacob and Furman 2001). Thus, it is important to identify the factors associated with persistent dizziness and unsteadiness after PCI. Nevertheless, relevant research is lacking at present.

Studies have shown that VBD may be correlated with dizziness and imbalance, especially BA tortuosity (Cosar et al. 2008; Huh et al. 2020). A literature review showed that symptoms related to the VIII cranial nerve were present in approximately 41% of patients with VBD (Gutierrez, Sacco, and Wright 2011). Another study showed that BA tortuosity was associated with undetermined isolated vertigo (Li et al. 2016). Therefore, it was speculated that BA tortuosity may affect the recovery of dizziness and unsteadiness after PCI. We aimed to assess the impact of BA tortuosity on the outcome and prognosis of dizziness and unsteadiness in PCI patients.

## 2 Methods

### 2.1 Participants

PCI inpatients presenting with dizziness and unsteadiness were prospectively and consecutively recruited from September 2021 to September 2022 in Beijing Tiantan Hospital. Inclusion criteria were as follows: age ≥ 18; acute PCI diagnosed by magnetic resonance imaging (MRI); admission within seven days after stroke onset; presented with dizziness and unsteadiness after stroke (mainly symptoms including vertigo, dizziness, vestibulo‐visual symptoms, and postural symptoms [Bisdorff et al. 2009]); National Institutes of Health Stroke Scale (NIHSS) ≤ 5 (Cucchiara et al. 2019). We recruited minor stroke patients (NIHSS ≤ 5) (Cucchiara et al. 2019) because moderate‐to‐severe neurological deficits may interfere with the assessment of dizziness and unsteadiness. Exclusion criteria were as follows: transient ischemic attack; PCI combined with anterior circulation infarction; prestroke mRS ≥ 2; inability to complete head MRI or poor image quality. This study was approved by the Medical Ethics Committee of Beijing Tiantan Hospital (KY 2021‐055‐02). All participants gave written informed consent.

### 2.2 Data Collection

#### 2.2.1 Baseline Data and Clinical Information

Collected baseline data included demographic information (gender and age), stroke risk factors (alcohol consumption, smoking, coronary heart disease, atrial fibrillation (AF), hyperlipidemia, diabetes, and hypertension), history of dizziness, prestroke mRS scores, and the administration of intravenous thrombolysis. AF included both a history of AF and newly diagnosed AF. History of dizziness was defined as having any vestibular symptoms before stroke, regardless of the type or duration. The vertigo symptom scale (VSS) was used to assess symptoms and handicap related to dizziness. The NIHSS score and nystagmus were recorded by neurological examination. Hamilton depression scale (HAMD) and Hamilton anxiety scale (HAMA) were completed to assess whether patients had anxiety (HAMA ≥ 7) or depression (HAMD ≥ 8) on admission and at discharge. The use of antivertiginous drugs after stroke onset was recorded.

#### 2.2.2 Imaging Data

A 3.0‐Tesla MR scanner with a 32‐channel head coil (Philips Ingenia, Best, Netherlands) was utilized to perform all MRI scans. MRI sequences contained T1‐weighted (T1W), T2‐weighted (T2W), and fluid‐attenuated inversion recovery sequence (FLAIR) images, diffusion‐weighted imaging (DWI), apparent diffusion coefficient (ADC), and three‐dimensional time‐of‐flight magnetic resonance angiography (3D‐TOF‐MRA). Detailed imaging parameters of the sequences are listed in Table S1. The analysis of images was conducted independently by two professional neuroradiologists blinded to clinical and outcome information.

Location of PCI was identified on DWI/ADC or T2W/FLAIR images. Posterior circulation was divided into proximal (medulla oblongata and posterior inferior cerebellar), middle (pons and anterior inferior cerebellar), and distal (midbrain, upper cerebellar, thalamus, and occipital lobe) territories (Chaves et al. 1994). Brainstem compression and VIII cranial nerve compression were identified on T2W images.

Smoker's criteria were used to evaluate the morphology of BA (Smoker et al. 1986). BA ectasia was defined as the maximum diameter of BA above 4.5 mm. The lateral displacement of the BA was scored as 0 (midline throughout), 1 (medial to the lateral margin of the clivus or dorsum sellae), 2 (lateral to the lateral margin of the clivus or dorsum sellae), and 3 (at the cerebellopontine angle cistern). The height of BA bifurcation was scored as 0 (at or below the dorsum sellae), 1 (within the suprasellar cistern), 2 (at the level of the third ventricle floor), and 3 (indenting and elevating the floor of the third ventricle). Moderate‐to‐severe BA tortuosity or bifurcation was defined to be a degree ≥ 2. The actual length of the BA was measured from the union of both vertebral arteries (VA) to the top of the BA along the arterial centerline in anteroposterior view of MRA. The straight‐line length of the vessel was defined as the shortest distance between the same two points. The BA tortuosity index (BATI) was calculated by the formula ([actual distance/straight line distance − 1] × 100) (Morris et al. 2011).

Vertebral artery dominance (VAD) was defined to be bilateral VA diameter difference ≥ 0.3 mm (Hong et al. 2009). The degree of intracranial stenosis was quantified according to the Warfarin‐Aspirin Symptomatic Intracranial Disease (WASID) study (Samuels et al. 2000). An ultrasonography examination was performed to estimate the extracranial part of the VA. Stenosis was considered significant when > 50%. Incomplete circle of Willis (CoW) was considered when any CoW vessel was absent or below 0.8 mm in diameter (Krabbe‐Hartkamp et al. 1998).

#### 2.2.3 Three‐Month Follow‐up

Patients were followed up at 3 months after PCI and categorized into dizziness‐unsteadiness group (DU group) and non–dizziness‐unsteadiness group (non‐DU group) based on the persistence or absence of dizziness and unsteadiness. The use of antivertiginous drugs was also recorded. The mRS score was completed to assess the recovery of neurological function, and a good functional outcome was defined as a score of 0–1 (Gardener et al. 2022; Hobeanu et al. 2022). The Dizziness Handicap Inventory (DHI) was completed to evaluate the living disability caused by dizziness. The total DHI index is 0–30 for mild disability, 31–60 for moderate disability, and 61–100 for severe disability (Jacobson and Newman 1990; Whitney et al. 2004). The activities‐specific balance confidence (ABC) scale was completed to evaluate the balance function. The ABC score < 67% was considered to have a high risk of falls (Lajoie and Gallagher 2004). The three‐level, five‐dimension EuroQol (EQ‐5D‐3L) was completed to describe life quality. Health status was converted into the EQ‐5D value based on the time trade‐off (TTO) Chinese value set (Zhuo et al. 2018).

### 2.3 Statistical Analysis

Statistical analysis was performed using SPSS 26.0 software (IBM). Continuous variables were indicated by mean ± standard deviation (SD) or median (interquartile range, IQR). Intergroup comparison was summarized as a *t*‐test or Mann‐Whitney non‐parametric test. Categorical variables were compared and summarized as χ^2^ test. To investigate the independent risk factors for dizziness and unsteadiness after PCI, the baseline parameters were first subjected to the univariate analysis. Then, the parameters with *p* values of < 0.10 were included in the binary logistic regression analysis. The odds ratio (OR) and 95% confidence interval (CI) were computed for each variable. A *p* value of < 0.05 was considered statistically significant.

## 3 Results

### 3.1 Baseline Characteristics

**FIGURE 1:**
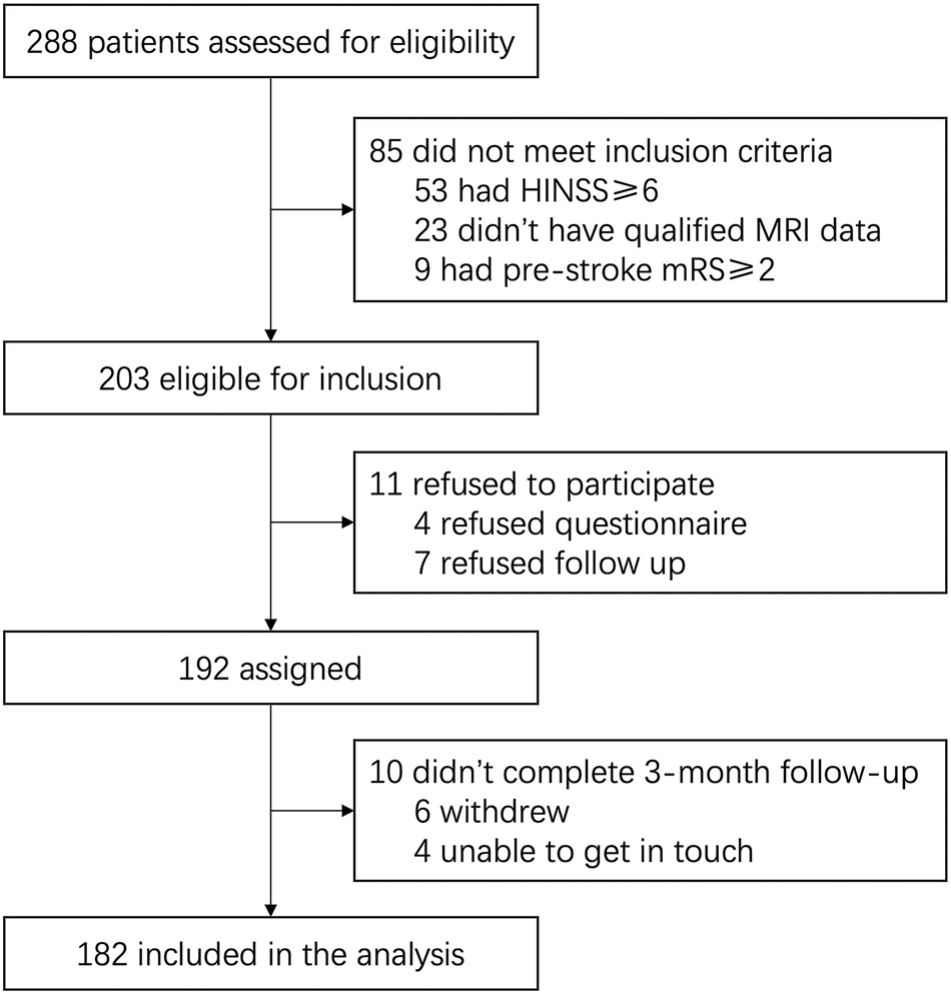
Study flow chart.

A total of 288 patients who had acute PCI and presented with dizziness and unsteadiness were recruited. Among them, 85 patients did not meet the inclusion criteria, 11 refused to participate, and 10 did not complete 3‐month follow‐up and were therefore excluded. Finally, 182 patients (84.6% male, mean age 58.17 ± 11.67) were included for analysis (Figure 1, Table 1). The proximal territory of the posterior circulation was the most affected segment (87/182, 47.8%). Ninety‐seven patients (53.3%) had BA tortuosity, including 19 (10.4%) with moderate‐to‐severe BA tortuosity.

**TABLE 1:** Baseline characteristics.

Baseline characteristics	Total (*n* = 182)	DU group (*n* = 58)	Non‐DU group (*n* = 124)	*p*‐value
Age, years old (SD)	58.17 ± 11.67	58.45 ± 10.35	58.03 ± 12.28	0.823
Male, *n* (%)	154 (84.6)	51 (87.9)	103 (83.1)	0.396
Risk factors				
Smoking history, *n* (%)	120 (65.9)	40 (69.0)	80 (64.5)	0.555
Drinking history, *n* (%)	107 (58.8)	36 (62.1)	71 (57.3)	0.539
Hypertension, *n* (%)	126 (69.2)	39 (67.2)	87 (70.2)	0.691
Diabetes, *n* (%)	63 (34.6)	23 (39.7)	40 (32.3)	0.328
Hyperlipidemia, *n* (%)	39 (21.4)	9 (15.5)	30 (24.2)	0.184
Coronary heart disease, *n* (%)	31 (17.0)	12 (20.7)	19 (15.3)	0.369
Atrial fibrillation, *n* (%)	18 (9.9)	7 (12.1)	11 (8.9)	0.501
History of stroke, *n* (%)	32 (17.6)	11 (19.0)	21 (16.9)	0.737
History of anxiety and/or depression, *n* (%)	1 (0.5)	0 (0)	1 (0.8)	1.000
History of dizziness, *n* (%)	27 (14.8)	11 (19.0)	16 (12.9)	0.284
Prestroke mRS, median (IQR)	0 (0*–*0)	0 (0*–*0)	0 (0*–*0)	0.986
NIHSS on admission, median (IQR)	2 (1*–*3)	2 (1*–*4)	2 (1*–*3)	0.594
Thrombolysis, *n* (%)	27 (14.8)	3 (5.2)	24 (19.4)	0.012^*^
Antivertiginous drug use, *n* (%)	21 (11.5)	8 (13.8)	13 (10.5)	0.515
Stroke lesion				
Proximal territory, *n* (%)	87 (47.8)	35 (60.3)	52 (41.9)	0.021^*^
Middle territory, *n* (%)	50 (27.5)	12 (20.7)	38 (30.6)	0.161
Distal territory, *n* (%)	29 (15.9)	7 (12.1)	22 (17.7)	0.330
Multiterritory, *n* (%)	16 (8.8)	4 (6.9)	12 (9.7)	0.537
Tinnitus, *n* (%)	25 (13.7)	10 (17.2)	15 (12.1)	0.347
Nystagmus				
Horizontal unidirectional nystagmus, *n* (%)	48 (26.4)	17 (29.3)	31 (25.0)	0.539
Horizontal direction‐changing nystagmus, *n* (%)	18 (9.9)	9 (15.5)	9 (7.3)	0.082
Rotary nystagmus, *n* (%)	3 (1.6)	2 (3.4)	1 (0.8)	0.239
Vertical nystagmus, *n* (%)	8 (4.4)	4 (6.9)	4 (3.2)	0.268
VSS score, median (IQR)	6 (5–8)	7 (5–9)	6 (5–7)	0.023^*^
Anxiety and depression				
Anxiety on admission, *n* (%)	20 (11.0)	8 (13.8)	12 (9.7)	0.408
Depression on admission, *n* (%)	29 (15.9)	11 (19.0)	18 (14.5)	0.445
Anxiety at discharge, *n* (%)	23 (12.6)	7 (12.1)	16 (12.9)	0.875
Depression at discharge, *n* (%)	25 (13.7)	9 (15.5)	16 (12.9)	0.633
Vertebrobasilar artery features				
VBD, *n* (%)	33 (18.1)	10 (17.2)	23 (18.5)	0.831
BA diameter, mm (SD)	3.61 ± 0.74	3.61 ± 0.80	3.61 ± 0.71	0.963
BA tortuosity, score ≥ 2, *n* (%)	19 (10.4)	12 (20.7)	7 (5.6)	0.002^*^
BATI^a^, median (IQR)	5.03 (1.94–10.14)	7.96 (2.92–15.18)	4.47 (1.69–8.62)	0.005^*^
BA bifurcation, score ≥ 2, *n* (%)	104 (57.1)	36 (62.1)	68 (54.8)	0.358
VAD, *n* (%)	87 (47.8)	26 (44.8)	61 (49.2)	0.583
Compression				
Brainstem compression, *n* (%)	11 (6.0)	7 (12.1)	4(3.2)	0.039^*^
VIII cranial nerve compression, *n* (%)	6 (3.3)	5(8.6)	1 (0.8)	0.013^*^
Incomplete CoW, *n* (%)	143 (78.6)	46 (79.3)	97 (78.2)	0.868
Significant stenosis > 50%				
BA, *n* (%)	24 (13.2)	9 (15.5)	15 (12.1)	0.525
Intracranial VA, *n* (%)	68 (37.4)	24 (41.4)	44 (35.5)	0.444
Extracranial VA, *n* (%)	60 (33.0)	23 (39.7)	37 (29.8)	0.189
VBA, *n* (%)	91 (50.0)	32 (55.2)	59 (47.6)	0.340

Abbreviations: BA, basilar artery; BATI, basilar artery tortuosity index; CoW, circle of Willis; DU group: dizziness‐unsteadiness group; mRS, modified Rankin Scale; NIHSS, National Institutes of Health Stroke Scale; VA, vertebral artery; VAD, vertebral artery dominance; VBA, vertebrobasilar artery; VBD, vertebrobasilar dolichoectasia; VSS, vertigo symptom scale.

^a^ Tortuosity index was calculated by the formula ([actual distance/straight line distance ‐ 1] × 100).

* Significant statistical difference according to *p* < 0.05.

**FIGURE 2:**
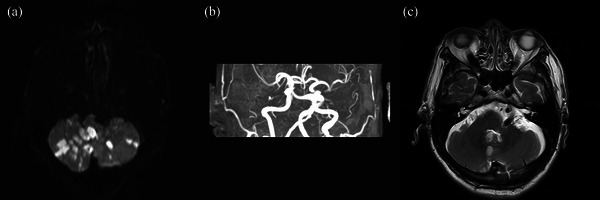
A male patient aged 70 years and complaining of “dizziness, nausea, unsteadiness, and tinnitus.” (a) DWI shows multiple infarcts in the bilateral cerebellar hemisphere. (b) MRA shows VBD. (c) Axial T2‐weighted images show that tortuous basilar artery compresses and pulls on the pons and VIII cranial nerve.

At the 3‐month follow‐up, 58 (31.9%) patients reported persistent dizziness and unsteadiness. Among them, 12 (20.7%) had moderate‐to‐severe BA tortuosity (see Figure 2 for a typical case). Univariate analysis showed that moderate‐to‐severe BA tortuosity (*p* = 0.004), BATI (*p* = 0.004), lesions involving the proximal posterior circulation territory (*p* = 0.022), thrombolysis (*p* = 0.020), brainstem compression (*p* = 0.029), VIII cranial nerve compression (*p* = 0.027), and horizontal direction‐changing nystagmus (*p* = 0.089) tended to be associated with dizziness and unsteadiness in 3 months after stroke (Table S2). The use of antivertiginous drugs was comparable between the two groups (*p* = 0.516). The types of drugs were as follows: 15 patients received betahistine hydrochloride, 3 received difenidol, 2 received diphenhydramine, and 1 received diazepam.

### 3.2 Clinical Outcome of DU Group

Patients in the DU group had higher mRS scores and a lower proportion of good functional outcome (*p* < 0.001). Approximately 36.2% (21/58) of patients in the DU group had moderate‐to‐severe living disability due to dizziness, and 27.6% (16/58) had a high risk of falls (*p* < 0.001). The EQ‐5D‐3L value was also significantly lower in the DU group (*p* < 0.001), with significant moderate‐to‐severe problems in most dimensions (Table 2).

**TABLE 2:** Functional outcome, risk of falls, and quality of life between the two groups.

	DU group (*n* = 58)	Non‐DU group (*n* = 124)	*p*‐value
mRS, median (IQR)	1 (1–2)	1 (0–1)	< 0.001
Good functional outcome, *n* (%)	36 (62.1)	113 (91.1)	< 0.001
VSS score, median (IQR)	8 (6–12)	*—*	*—*
DHI score, median (IQR)	20 (8–38)	*—*	*—*
Mild disability, *n* (%)	37 (63.8)	*—*	*—*
Moderate disability, *n* (%)	14 (24.1)	*—*	*—*
Severe disability, *n* (%)	7 (12.1)	*—*	*—*
High risk of falls^a^, *n* (%)	16 (27.6)	3 (2.4)	< 0.001
EQ‐5D‐3L value in 3 months, median (IQR)	0.96 (0.89–1.00)	1.00 (1.00–1.00)	< 0.001
Mobility level ≥ 2, *n* (%)	21 (36.2)	7 (5.6)	< 0.001
Self‐care level ≥ 2, *n* (%)	3 (5.2)	2 (1.6)	0.329
Level of usual activities ≥ 2, *n* (%)	28 (48.3)	8 (6.5)	< 0.001
Pain/discomfort level ≥ 2, *n* (%)	20 (34.5)	8 (6.5)	< 0.001
Anxiety/depression level ≥ 2, *n* (%)	17 (29.3)	16 (12.9)	0.007

Abbreviations: DHI, dizziness handicap inventory; EQ‐5D‐3L, three‐level five‐dimension EuroQol; mRS, modified Rankin Scale; VSS, vertigo symptom scale.

^a^ ABC score < 67% was considered to have a high risk of falls.

### 3.3 Logistic Regression Analysis of Factors Related to Persistent Dizziness and Unsteadiness After PCI

**FIGURE 3:**
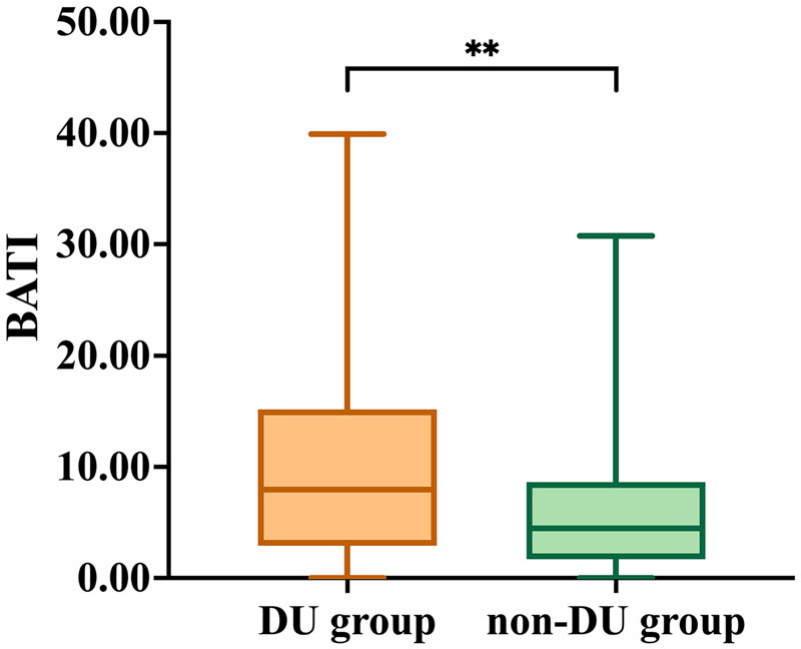
The Box plot shows the comparison of basilar artery tortuosity index (BATI) between DU group and non‐DU group. BATI was calculated by the formula ([actual distance/straight line distance −1] × 100). ** Represents *p *< 0.01.

Binary logistic regression analysis identified moderate‐to‐severe BA tortuosity (OR, 4.474; 95% CI, 1.591–12.579; *p* = 0.004) and lesions involving the proximal posterior circulation territory (OR, 2.146; 95% CI, 1.097–4.199; *p* = 0.026) as risk factors for persistent dizziness and unsteadiness in 3 months after stroke, while thrombolysis (OR, 0.280; 95% CI, 0.079–0.992; *p* = 0.049) as a protective factor (Table 3, model 1). We further introduced BATI into binary logistic regression analysis and found that BATI was also significantly associated with persistent dizziness and unsteadiness after stroke (OR, 1.072; 95% CI, 1.028–1.119; *p* = 0.001) (Table 3, model 2). Box plots were used to analyze the associations between BATI and dizziness and unsteadiness after stroke (Figure 3). These associations remained statistically significant after adjusting for gender, age, relevant clinical variables, and MRI parameters.

**TABLE 3:** Factors associated with dizziness and unsteadiness in 3 months after stroke.

Models	OR (95% CI)	*p*‐value
Model 1 (with BA tortuosity)		
BA tortuosity, score ≥ 2	4.474 (1.591*–*12.579)	0.004
Stroke in the proximal territory	2.146 (1.097*–*4.199)	0.026
Thrombolysis	0.280 (0.079*–*0.992)	0.049
Model 2 (with BATI)		
BATI^a^	1.072 (1.028*–*1.119)	0.001
Stroke in the proximal territory	2.164 (1.103*–*4.246)	0.025
Thrombolysis	0.184 (0.048*–*0.705)	0.013

Abbreviations: BA, basilar artery; BATI, basilar artery tortuosity index.

^a^ Tortuosity index was calculated by the formula ([actual distance/straight line distance − 1] × 100).

## 4 Discussion

To the best of our knowledge, this prospective study is the first to investigate the impact of BA morphological parameters on the outcome of dizziness and unsteadiness after PCI. The main finding was that nearly one‐third of patients who develop dizziness after PCI experienced persistent dizziness and unsteadiness 3 months after stroke, with significantly high risk of falls and decreased quality of life. Both moderate‐to‐severe BA tortuosity and BATI had the value of predicting the persistence of dizziness and unsteadiness after PCI. Stroke in the proximal posterior circulation territory also acted as a predictive factor, while thrombolysis had a negative impact.

### 4.1 BA Tortuosity and Dizziness/Unsteadiness

The association between BA tortuosity and dizziness/unsteadiness in patients with PCI may be ascribed to multiple mechanisms. On the one hand, the mechanical compression of vestibular structures by a tortuous BA can cause vestibular dysfunction. Studies indicated that the compression of vestibular structures or their connections, such as the VIII cranial nerve and inferior brainstem, can result in their angulation, displacement, or deformity and subsequently lead to dizziness and unsteadiness (Cosar et al. 2008; Huh et al. 2020; Holmes, Kerezoudis, and Mustafa 2022). Furthermore, chronic stimulation of the VIII cranial nerve by vascular compression will cause continuous excitation of the vestibular nucleus, which results in its reorganization and affects vestibular compensatory function (Lacour, Helmchen, and Vidal 2016). Since compression is always progressive and leaves room for compensation, some patients may be asymptomatic (Kierig et al. 2023; Lou and Caplan 2010). However, the occurrence of PCI may disrupt the compensatory balance, which leads to prolonged symptoms. In our study, we found more brainstem and VIII cranial nerve compression in the DU group, which is consistent with these findings.

On the other hand, the hemodynamic changes of posterior circulation may also play a key role. The more severe the tortuosity is, the more severe the hemodynamic disorder will be (Wang et al. 2022). Studies based on transcranial Doppler ultrasound have shown a decreased BA blood flow velocity in approximately half of patients with VBD (Kumral et al. 2005). FLAIR vascular hyperintensity (FVH), as a marker of slow blood flow, was observed in patients with ischemic stroke and VBD (Förster et al. 2015). Moreover, the tortuous BA will lead to the distortion of the perforator artery via mechanical traction, which results in slow and reduced blood flow (Lou and Caplan 2010). These factors may lead to the hypoperfusion of posterior circulation, which impairs the connections between cerebellum, brainstem, and vestibular cortex, induces vestibular dysfunction, and thus impedes the recovery process of dizziness and unsteadiness after stroke (Li et al. 2016).

Considering the potential impact of history of dizziness, VAD, and significant stenosis of the VBA (Li et al. 2016), we adjusted these variables in binary logistic regression. The results indicated that both moderate‐to‐severe BA tortuosity and BATI were still independently associated with persistent dizziness and unsteadiness after PCI.

### 4.2 Proximal Territory Stroke and Dizziness/Unsteadiness

In this study, the location of stroke also had a predominant effect on dizziness and unsteadiness after PCI, which is consistent with previous reports. Studies have indicated that patients with PCI in the proximal territory experienced more severe dizziness and imbalance and a prolonged compensatory course (Zwergal et al. 2020; Baier et al. 2015). In patients with lateral medullary infarction, disequilibrium was the most common symptom at 12‐month follow‐up, followed by dysarthria and dizziness (Nelles et al. 1998). This is associated with damage to central vestibular structures such as vestibular nuclei and cerebellum, which are crucial for generating, processing, and transmitting vestibular signals (Kim et al. 2022). Lesions in these areas may affect the processing of central vestibular compensation, which results in a slower recovery of dizziness and unsteadiness (Baier et al. 2015).

### 4.3 Thrombolysis and Dizziness/Unsteadiness

In this study, thrombolysis was a protective factor for persistent dizziness and unsteadiness after PCI. Intravenous thrombolysis (IVT) is the recommended therapy in acute ischemic stroke, and its effectiveness has been widely demonstrated (Emberson et al. 2014). But the decision of IVT in minor PCI patients with dizziness/unsteadiness remains considerably uncertain, mainly because these symptoms are generally not considered to be disabling (Lee and Kim 2015; Powers et al. 2019). However, in our study, nearly one‐third of our patients still had persistent dizziness and unsteadiness at 3‐month follow‐up. This not only poses a substantial risk for falls but also hinders their mobility, usual activities, and quality of life, which is consistent with previous reports (Curthoys and Halmagyi 1995; Balaban and Jacob 2001; Jacob and Furman 2001). Therefore, a recent study indicated that these symptoms should be regarded as disabling symptoms (Machner et al. 2021). The results of our study preliminarily suggest that thrombolysis may improve the outcome of dizziness and unsteadiness after minor PCI. However, since not all patients arrived at the emergency department within 4.5 h after stroke onset, this finding should be interpreted with caution. Future studies could focus on the decision‐making of thrombolysis in these patients.

### 4.4 Clinical Management Strategies

Limited reports are available regarding the management of dizziness and unsteadiness associated with VBD or BA tortuosity. A literature review recommended conservative treatment and long‐term follow‐up, which highlights the importance of rehabilitation exercises and fall prevention (Frosolini et al. 2023). A recent meta‐analysis revealed that vestibular rehabilitation therapy (VRT) could significantly improve the balance and gait function of stroke patients (Meng et al. 2023). Therefore, based on our preliminary study, we suggest that early initiation of VRT in patients with proximal territory PCI and significant BA tortuosity is recommended to mitigate the risk of falls and improve patient outcomes.

### 4.5 Limitations

There are several limitations that should be noted. First, this is a single‐center cohort study with a limited sample size. Second, only the short‐term prognosis of patients was analyzed in this study. Third, the manifestation of vestibular symptoms is subjective. Although we recorded nystagmus, VSS, DHI, and ABC scales to assess dizziness and unsteadiness, electronystagmography, caloric test, etc., were lacking, partially because patients cannot cooperate well with these examinations in the acute phase of stroke. Finally, the hemodynamics of the VBA were not evaluated, which may help elucidate the underlying mechanisms of BA tortuosity and dizziness.

## 5 Conclusion

In this prospective study, it was demonstrated that nearly one‐third of patients who develop dizziness after PCI experienced persistent dizziness and unsteadiness 3 months after stroke, with significantly high risk of falls and decreased quality of life. Prominent BA tortuosity was closely associated with persistent dizziness and unsteadiness after PCI. Lesions involving the proximal posterior circulation territory also acted as a predictive factor, while thrombolysis had a negative impact. It is crucial to optimize clinical management for these patients, including vestibular rehabilitation and fall prevention strategies.

## Author Contributions

**Jiashu Li**: investigation; formal analysis; writing–original draft. **Xuesong Bai**: investigation; formal analysis. **Gaifen Liu**: methodology; data curation; visualization. **Zhaoxia Li**: data curation; resources; conceptualization. **Yan Wang**: conceptualization; data curation; resources. **Ruile Fang**: conceptualization; data curation; resources. **Fei Peng**: methodology; software; validation. **Xuge Chen**: methodology; software; visualization. **Yi Ju**: project administration; writing–review and editing; supervision. **Xingquan Zhao**: project administration; writing–review and editing; supervision; funding acquisition.

## Ethics Statement

This study was approved by the Medical Ethics Committee of Beijing Tiantan Hospital (KY 2021‐055‐02). All participants gave written informed consent.

## Conflicts of Interest

The authors declare no conflicts of interest.

### Peer Review

The peer review history for this article is available at https://publons.com/publon/10.1002/brb3.70097.

## Supporting information




**Supplementary Table S1**. The detailed MRI scan parameters in the present study
**Supplementary Table S2**. Univariable analysis of factors associated with dizziness and unsteadiness in three months after stroke.

## Data Availability

Data are available upon reasonable request. All data are available to researchers on request for purposes of reproducing the results or replicating the procedure by directly contacting the corresponding author.
