# Supplementary material for: Basilar Artery Tortuosity Increases the Risk of Persistent Dizziness and Unsteadiness After Posterior Circulation Infarction
Source: Brain Behav. 2024 Oct 22;14(10):e70097. doi: 10.1002/brb3.70097 (PMC11494399; doi:10.1002/brb3.70097)
Supplement: Supplementary file 1 — Supplementary Table S1. The detailed MRI scan parameters in the present study Supplementary Table S2. Univariable analysis of factors associated with dizziness and unsteadiness in three months after stroke. [file BRB3-14-e70097-s001.docx]

**SUPPLEMENTARY MATERIAL**

**Journal name:** Brain and Behavior

**Title**: Basilar artery tortuosity increases the risk of persistent dizziness and unsteadiness after posterior circulation infarction

**Supplementary Table S1. The detailed MRI scan parameters in the present study**

|  | TR (msec) | TE (msec) | FOV (mm) | Matrix (mm) | Flip angle (°) | Slice thickness (mm) | Voxel size (mm) | Time(min) |
| --- | --- | --- | --- | --- | --- | --- | --- | --- |
| 3D T1WI | 6.6 | 3.0 | 240×240×196 | 240×240 | 8 | - | 1.00×1.00×1.00 | 02:19 |
| T2WI | 4600 | 106 | 230×197×143 | 288×195 | 90 | 5 | 0.80×0.80×5.00 | 00:46 |
| 3D FLAIR | 4800 | 256 | 240×240×178 | 240×240 | - | - | 1.00×1.00×1.00 | 02:43 |
| DWI | 2227 | 58 | 184×226×143 | 116×109 | 90 | 5 | 1.60×2.04×5.00 | 00:20 |
| 3D TOF MRA | 22 | 3.5 | 200×200×81 | 384×250 | 18 | - | 0.52×0.80×1.35 | 01:54 |

MRI, magnetic resonance imaging; TR, repetition time; TE, echo time; FOV, field of view; 3D T1WI, three-dimensional T1-weighted imaging; T2WI, T2-weighted imaging; 3D FLAIR, three-dimensional fluid-attenuated inversion recovery sequence; DWI, diffusion-weighted imaging; 3D TOF-MRA, three-dimensional time-of-flight magnetic resonance angiography.

**Supplementary Table S2. Univariable analysis of factors associated with dizziness and unsteadiness in three months after stroke**

| Baseline characteristics | OR (95% CI) | p-value |
| --- | --- | --- |
| Age | 1.003 (0.977-1.030) | 0.822 |
| Male | 1.485 (0.593-3.723) | 0.399 |
| Risk factors |  |  |
| Smoking history | 1.222 (0.627-2.381) | 0.555 |
| Drinking history | 1.222 (0.645-2.314) | 0.539 |
| Hypertension | 0.873 (0.447-1.706) | 0.691 |
| Diabetes | 1.380 (0.723-2.635) | 0.329 |
| Hyperlipidemia | 0.576 (0.253-1.308) | 0.187 |
| Coronary heart disease | 1.442 (0.647-3.213) | 0.371 |
| Atrial fibrillation | 1.410 (0.517-3.847) | 0.502 |
| History of stroke | 1.148 (0.512-2.573) | 0.738 |
| History of anxiety and/or depression | 0.000 (0.000-Inf) | 0.988 |
| History of dizziness | 1.580 (0.682-3.662) | 0.286 |
| Pre-stroke mRS | 1.012 (0.468-2.186) | 0.976 |
| NIHSS on admission | 1.060 (0.876-1.284) | 0.548 |
| Thrombolysis | 0.227 (0.065-0.789) | 0.020* |
| Antivertiginous drug use | 1.366 (0.533-3.504) | 0.516 |
| Stroke lesion † |  |  |
| Proximal territory | 2.107 (1.116-3.978) | 0.022* |
| Middle territory | 0.590 (0.281-1.239) | 0.163 |
| Distal territory | 0.636 (0.255-1.588) | 0.333 |
| Multi-territory | 0.691 (0.213-2.244) | 0.539 |
| Tinnitus | 1.514 (0.635-3.611) | 0.350 |
| Nystagmus |  |  |
| Horizontal unidirectional nystagmus | 1.244 (0.620-2.496) | 0.539 |
| Horizontal direction-changing nystagmus | 2.347 (0.879-6.269) | 0.089 |
| Rotary nystagmus | 4.393 (0.390-49.459) | 0.231 |
| Vertical nystagmus | 2.222 (0.536-9.218) | 0.271 |
| VSS score | 1.072 (0.981-1.172) | 0.122 |
| Anxiety and depression |  |  |
| Anxiety on admission | 1.493 (0.575-3.879) | 0.410 |
| Depression on admission | 1.378 (0.604-3.145) | 0.446 |
| Anxiety at discharge | 0.926 (0.359-2.392) | 0.875 |
| Depression at discharge | 1.240 (0.512-3.000) | 0.634 |
| Vertebrobasilar artery features |  |  |
| VBD | 0.915 (0.404-2.073) | 0.831 |
| BA diameter | 1.010 (0.661-1.543) | 0.963 |
| BA tortuosity, score ≥ 2 | 4.360 (1.616-11.765) | 0.004* |
| BATI † | 1.058 (1.018-1.099) | 0.004* |
| BA bifurcation, score ≥ 2 | 1.348 (0.712-2.549) | 0.359 |
| VAD | 0.839 (0.449-1.569) | 0.583 |
| Compression |  |  |
| Brainstem compression | 4.118 (1.155-14.683) | 0.029* |
| VIII cranial nerve compression | 11.604 (1.323-101.736) | 0.027* |
| Incomplete CoW | 1.067 (0.496-2.293) | 0.868 |
| Significant stenosis > 50% |  |  |
| BA | 1.335 (0.547-3.258) | 0.526 |
| Intracranial VA | 1.283 (0.677-2.432) | 0.444 |
| Extracranial VA | 1.545 (0.805-2.965) | 0.191 |
| VBA | 1.356 (0.725-2.536) | 0.340 |

Asterisks mean a significant statistical difference according to the p-value < 0.05.

Abbreviations: DU group: dizziness-unsteadiness group; mRS, modified Rankin Scale; NIHSS, National Institutes of Health Stroke Scale; VSS, vertigo symptom scale; VBD, vertebrobasilar dolichoectasia; BA, basilar artery; BATI, basilar artery tortuosity index, VAD, vertebral artery dominance; CoW, circle of Willis; VA, vertebral artery; VBA, vertebrobasilar artery.

† Tortuosity index was calculated by the formula ([actual distance/straight line distance - 1] × 100).
